# Over-the-Scope Clip Closure of Persistent Gastrocutaneous Fistula After Percutaneous Endoscopic Gastrostomy Tube Removal: A Report of Two Cases

**DOI:** 10.7759/cureus.13206

**Published:** 2021-02-07

**Authors:** Shigenori Masaki, Keishi Yamada

**Affiliations:** 1 Surgery and Gastroenterology, Miyanomori Memorial Hospital, Sapporo, JPN; 2 Clinical Engineering, Miyanomori Memorial Hospital, Sapporo, JPN

**Keywords:** over-the-scope clip, otsc, fistula, gastrocutaneous fistula, percutaneous endoscopic gastrostomy, peg

## Abstract

Persistent gastrocutaneous fistulas have conventionally been treated surgically. Over-the-scope clip (OTSC) was developed as an endoscopic closure device for full-thickness gastrointestinal defects and has become one of the treatment options for gastrocutaneous fistula. Herein, we report two cases of gastrocutaneous fistulas treated using OTSC. Case 1 was a 71-year-old woman and case 2 was an 88-year-old man, both of whom had severe frailty and had a persistent gastrocutaneous fistula after removal of the percutaneous endoscopic gastrostomy (PEG) tube. OTSC closure was chosen over surgical closure to reduce invasiveness. In case 1, OTSC was deployed using a suction method, which was technically successful. However, the fistula reopened two days later, indicating clinical failure of the OTSC. The cause of the failure may be due to an inadequate suction of the fistula into the applicator cap. Based on the experience of OTSC failure in case 1, OTSC in case 2 was deployed using the Anchor to pull the fistula into the cap more reliably. Fistula did not recur during the 30-month follow-up, indicating the clinical success of OTSC in case 2. The use of Anchor may increase the success rate of OTSC, but there is a dilemma that the use of Anchor increases cost. In summary, OTSC has the advantage of being less invasive compared to conventional surgery; however, the application of OTSC for chronic fistulas remains challenging due to issues regarding clinical success rate and cost.

## Introduction

Gastrocutaneous fistula has conventionally been treated surgically [1]. One of the main causes of a gastrocutaneous fistula is the removal of the percutaneous endoscopic gastrostomy (PEG) tube [2]. Enteral nutrition via PEG is indicated in patients with dysphagia [3]. After the complete resumption of oral intake, the PEG tube can be removed [4]. Although the gastrostomy site closes spontaneously in most cases after the removal of the PEG tube, persistent gastrocutaneous fistula can occasionally occur [1,2]. Over-the-scope clip (OTSC) was developed as an endoscopic full-thickness gastrointestinal closure device [5] and has become one of the treatment options for gastrocutaneous fistula because it is less invasive compared to conventional surgical closure [6]. However, the clinical success rates of OTSC closure in chronic fistula have been reported to be 40%-50% [7,8].

The key to the success of OTSC closure is an adequate suction of the fistula into the applicator cap [6-8]. Tissue induration of the chronic fistula makes it difficult to suction the fistula adequately into the cap; therefore, aggressive use of accessory devices, such as the Anchor, is recommended [7]. However, cost increase due to the use of the Anchor should be taken into account; the OTSC system and Anchor cost 770 USD each. Application of OTSC in chronic fistulas still remains a challenge in terms of clinical success rate and cost [7]. Herein, we report two cases of unsuccessful and successful treatments using OTSC for persistent gastrocutaneous fistula after PEG tube removal.

## Case presentation

Case 1

A 71-year-old woman underwent PEG using a 20-Fr introducer PEG kit at the age of 65 years, due to severe dysphagia caused by disuse syndrome after an emergent surgery for necrotic ischemic colitis. She had a bipolar disorder and was severely frail. She gradually resumed her oral intake due to the effects of continuous rehabilitation, and finally the PEG tube was removed at six years after PEG. However, the gastrostomy site did not close spontaneously over a period of one month despite conservative medical management using a proton pump inhibitor. She was admitted to our hospital for the persistent gastrocutaneous fistula.

**Figure 1:**
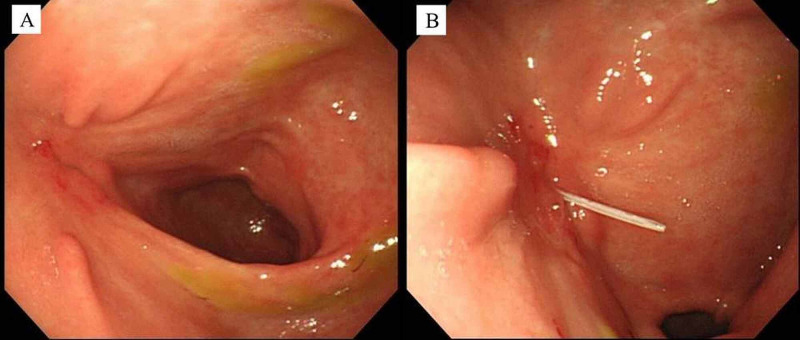
Endoscopic views before OTSC closure (case 1) (A) The gastrocutaneous fistula was located on the anterior wall of the antrum. (B) A plastic cannula passing through the fistula from the body surface.

On admission to our hospital, her height and weight were 155 cm and 35 kg, respectively. Body mass index (BMI) was 14.6. Severe dermatitis was observed around the gastrostomy site. Esophagogastroduodenoscopy (EGD) on day 1 revealed a gastrocutaneous fistula on the anterior antral wall (Figure 1).

**Figure 2:**
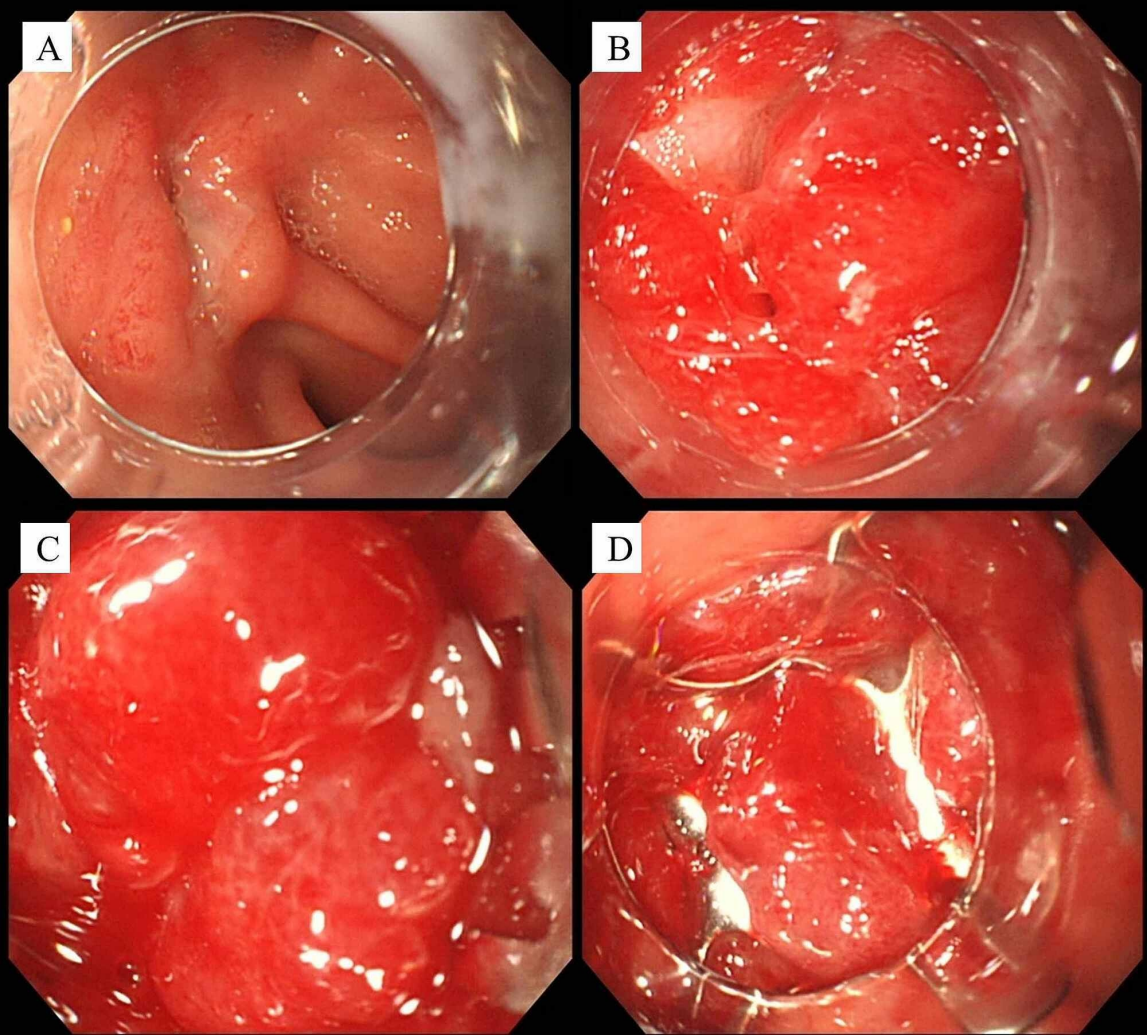
Endoscopic views during OTSC closure (case 1) (A) The fistula of the antrum. (B,C) Suction of the fistula into the applicator cap. (D) Deployment of OTSC.

The size of the fistula measured by endoscopy was 6 mm. OTSC closure was chosen over surgical closure considering the patient’s general condition. On day 4, OTSC closure (size 9 mm, t-type) using the suction method was performed (Figure 2).

**Figure 3:**
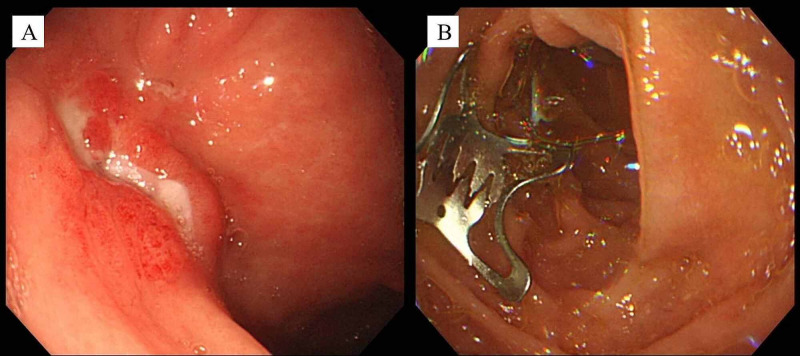
Endoscopic views at 4 days after OTSC closure (case 1) (A) The OTSC dropping off from the fistula. (B) OTSC advanced to the duodenum.

A GIF-Q260J endoscope (Olympus, Tokyo, Japan) was used for this procedure. Following the OTSC closure, leakage of gastric juice from the fistula stopped after the OTSC closure but started again two days later. EGD on day 8 revealed the OTSC dropping off from the fistula, which was endoscopically removed (Figure 3).

A second attempt for OTSC or surgical closure of the fistula was not performed considering the invasiveness of the procedure and further increase in the medical cost. Instead, PEG was performed through the same fistula as the patient still had mild dysphagia and was concerned about the exacerbation of the symptom due to the underlying condition. No skin problems were observed after the PEG procedure. On day 18, the patient was discharged and transferred to the long-term care hospital where she was originally hospitalized.

Case 2

An 88-year-old man underwent PEG using a 20-Fr introducer PEG kit at the age of 86 years due to severe dysphagia caused by disuse syndrome following a surgery for right femoral neck fracture. He had severe dementia and was severely frail. He resumed complete oral intake following rehabilitation, and the PEG tube was removed at two years after PEG. However, the gastrostomy site did not close spontaneously over a period of three weeks despite conservative medical management using a proton pump inhibitor. He was admitted to our hospital for the persistent gastrocutaneous fistula.

**Figure 4:**
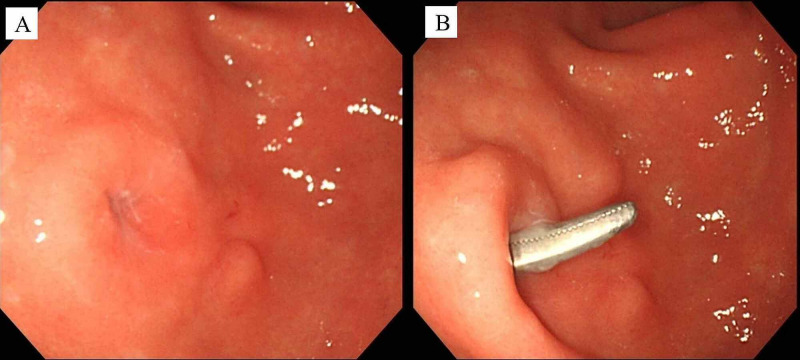
Endoscopic views before OTSC closure (case 2) (A) The fistula located on the antrum. (B) Pean forceps passing through the fistula from the body surface.

On admission to our hospital, his height and weight were 148 cm and 44 kg, respectively, with a BMI of 20.1. Severe dermatitis around the gastrostomy site was observed. EGD on day 2 revealed a gastrocutaneous fistula on the anterior antral wall. Pean forceps inserted through the external opening of the fistula were observed in the stomach on endoscopy (Figure 4).

**Figure 5:**
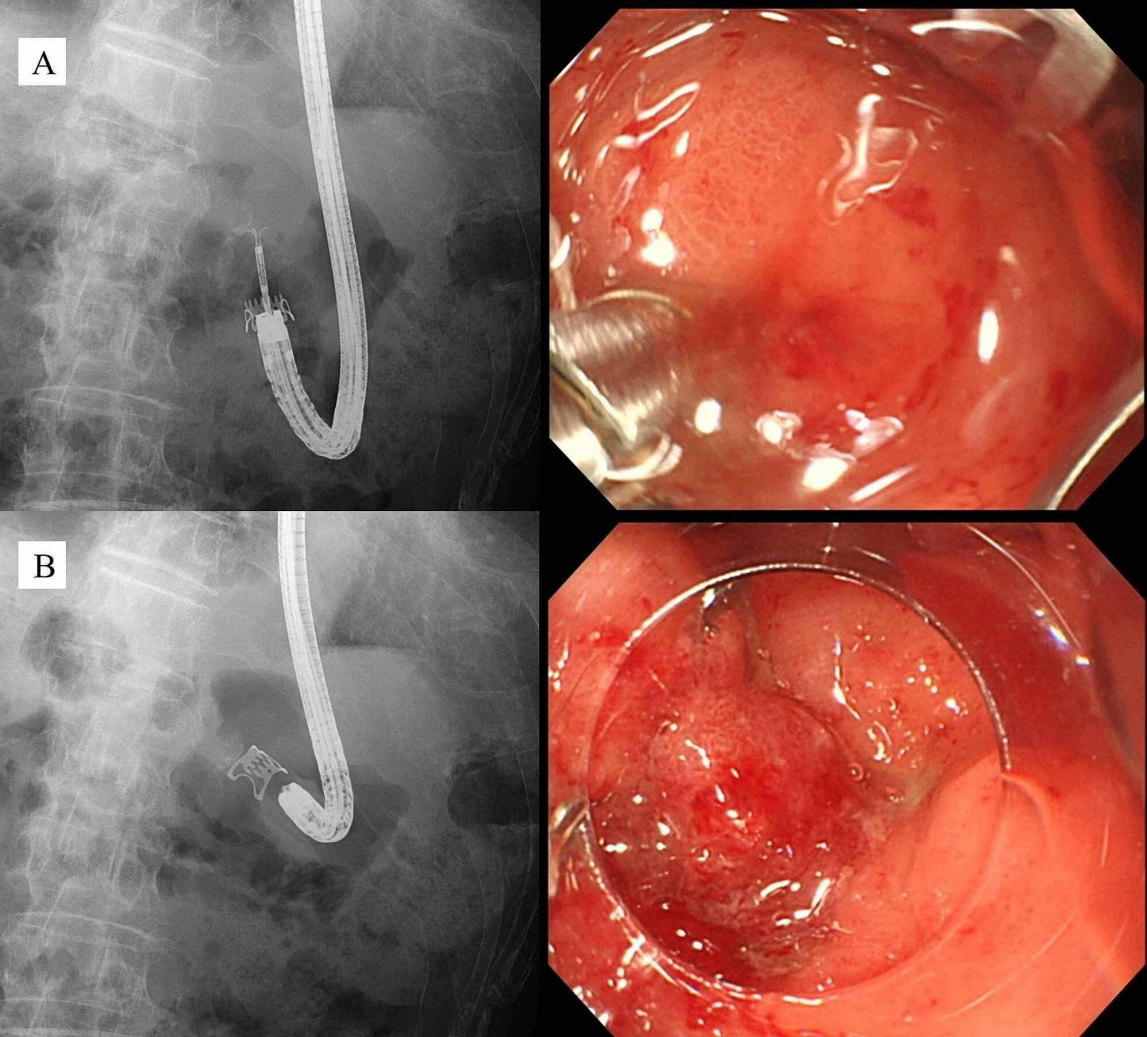
Fluoroscopic and endoscopic views during OTSC closure (case 2) (A) Anchor insertion into the fistula and retraction of the fistula into the applicator cap. (B) Completion of OTSC deployment.

The size of the fistula measured by endoscopy was 6 mm. On day 7, OTSC closure (size 9 mm, t-type) was attempted using the suction method with digital pressure on the external opening of the fistula to lead the internal opening into the applicator cap, but it was difficult to adequately suction the fistula into the cap due to tissue induration. Therefore, the Anchor was used to pull the fistula into the cap and the OTSC was deployed (Figure 5).

Leakage of gastric juice from the fistula stopped after OTSC closure. On day 18, he was discharged from our hospital and returned to the nursing home. No recurrence of the fistula was observed during the 30-month follow-up.

## Discussion

In this report, we have described unsuccessful and successful cases of gastrocutaneous fistulas following PEG tube removal managed using OTSC. The mean rates of clinical success of OTSC in chronic fistula have been reported to be approximately 40%-50% [7-12]. Although our report focused on only two cases, our results were consistent with those of previous reports. The causes of failure of OTSC in fistula are fibrosis and poor extensibility of the fistula [7,10-12]. The key to successful treatment is overcoming the difficulty in adequately suctioning the indurated tissue of the fistula into the applicator cap.

In case 1, OTSC was deployed after confirming adequate suction of the fistula into the cap during the procedure. Although the OTSC closure was considered to be successful, it was a clinical failure. Therefore, the suction of the fistula into the cap may not have been adequate due to fibrotic induration of the tissue. Nearly 50% of patients with the initial technical success of OTSC have been reported to have a recurrence during the subsequent follow-up [13]. Even when the OTSC closure appears as technically successful, careful observation is necessary due to potential recurrence of the fistula.

In case 2, the OTSC closure using the Anchor was clinically successful. The Anchor is an accessory device with three hooks for pulling the fistula up into the applicator cap [7,10,11,14]. Insufficient suction of the indurated tissue of the fistula into the cap causes failure of OTSC closure; therefore, the aggressive use of an accessory device is recommended for successful long-term closure of the fistula [7,11]. The frequency of the use of the Anchor in fistula closure using the OTSC is reported to be approximately 8%-50% [2,7,9,11,12], and the success rate of OTSC closure using the Anchor is reported to be approximately 50% [7,12]. The modest success rate of OTSC closure using the Anchor may be due to the fact that the application of the Anchor to cases where suction of the fistula into the cap is difficult owing to a high degree of tissue induration. In this case, the use of the Anchor allowed reliable retraction of the fistula into the cap, leading to a successful procedure. Although the Anchor is useful, the involved cost can be a barrier in its application [7]. For example, the cost of the OTSC system including the Anchor is approximately 1540 USD (OTSC system 770 USD; Anchor 770 USD) in Japan. In contrast, the medical treatment fee for OTSC closure of gastrocutaneous fistula is 990 USD. Furthermore, there is no insurance reimbursement for OTSC devices. Therefore, in hospitals in Japan, if OTSC closure for fistula is performed using the Anchor, a cost of at least 550 USD is incurred. Therefore, the Anchor must be judiciously used despite its usefulness.

In both cases, the fistula was formed from a 20-Fr PEG tube. The fistula size was endoscopically measured as 6 mm in both cases, with reference to the thickness of the Pean forceps inserted through the fistula from the body surface into the stomach. A foreign body retrieval grasper has been reportedly used for measuring the size of the fistula [6]. The success rate of OTSC closure is reported to be relatively better in fistulas with small sizes (≥10 mm); therefore, precise measurement of the fistula size and the choice of adequate size of OTSC are important in achieving clinical success [10]. Comparing the patient background factors between cases 1 and 2, both patients were old and severely frail and had similar fistula sites and sizes; however, the period from PEG to removal was different between the two cases (case 1, six years versus case 2, two years). Longer duration of PEG tube placement is reported to be a risk factor of the development of persistent fistula [15]. The failure and success of OTSC closure in cases 1 and 2 may have been associated with the duration of PEG tube placement.

Induration and epithelialization of the tract are well known to diminish fistula healing [16]. Various attempts for de-epithelialization to promote fistula healing, such as surgical excision [16], argon plasma coagulation [17], and endoscopic submucosal dissection [18], have been reported. Conversely, it has been reported that thermal injury and mechanical trauma to the fistula may not contribute to OTSC use due to its transmural closure effects [11]. Additionally, tissue edema due to thermal damage can make it difficult to grasp the tissue into the cap [11,19]. In our case series, a de-epithelialization procedure was not applied considering the lack of standardized procedure with sufficient evidence. Currently, the addition of the de-epithelialization procedure before OTSC deployment is dependent on the discretion of the endoscopist [19].

In the case of an unsuccessful OTSC closure for fistula, the next strategy is controversial. Repeated OTSC attempts can be considered, but the cost is a concern. The next line of treatment including endoscopic closure or surgery must be decided on a case-to-case basis according to the feasibility of the treatment at the hospital and considering the patient’s general and financial conditions.

## Conclusions

OTSC closure can be one of the treatment options for persistent gastrocutaneous fistula after PEG tube removal. The key to successful OTSC closure is adequate suction of the fistula into the applicator cap. Precise measurement of the fistula size and the choice of adequate size of OTSC are important in achieving successful OTSC closure. Longer duration of PEG tube placement may be associated with unsuccessful OTSC closure. The use of an Anchor was found to be helpful for successful OTSC closure. However, issues regarding the clinical success rate, cost, and subsequent strategy in case of OTSC closure failure are unresolved.

## References

[1] Bender JS, Levison MA (1991). Complications after percutaneous endoscopic gastrostomy removal. Surg Laparosc Endosc.

[2] Haito-Chavez Y, Law JK, Kratt T (2014). International multicenter experience with an over-the-scope clipping device for endoscopic management of GI defects (with video). Gastrointest Endosc.

[3] Westaby D, Young A, O'Toole P, Smith G, Sanders DS (2010). The provision of a percutaneously placed enteral tube feeding service. Gut.

[4] Wilmskoetter J, Herbert TL, Bonilha HS (2017). Factors associated with gastrostomy tube removal in patients with dysphagia after stroke. Nutr Clin Pract.

[5] Kirschniak A, Kratt T, Stüker D, Braun A, Schurr MO, Königsrainer A (2007). A new endoscopic over-the-scope clip system for treatment of lesions and bleeding in the GI tract: first clinical experiences. Gastrointest Endosc.

[6] Singhal S, Changela K, Culliford A, Duddempudi S, Krishnaiah M, Anand S (2015). Endoscopic closure of persistent gastrocutaneous fistulae, after percutaneous endoscopic gastrostomy (PEG) tube placement, using the over-the-scope-clip system. Therap Adv Gastroenterol.

[7] Kobara H, Mori H, Fujihara S (2017). Outcomes of gastrointestinal defect closure with an over-the-scope clip system in a multicenter experience: an analysis of a successful suction method. World J Gastroenterol.

[8] Lee HL, Cho JY, Cho JH (2018). Efficacy of the over-the-scope clip system for treatment of gastrointestinal fistulas, leaks, and perforations: a Korean multi-center study. Clin Endosc.

[9] Morrell DJ, Winder JS, Johri A (2020). Over-the-scope clip management of non-acute, full-thickness gastrointestinal defects. Surg Endosc.

[10] Kobara H, Mori H, Nishiyama N (2019). Over-the-scope clip system: a review of 1517 cases over 9 years. J Gastroenterol Hepatol.

[11] Baron TH, Song LM, Ross A, Tokar JL, Irani S, Kozarek RA (2012). Use of an over-the-scope clipping device: multicenter retrospective results of the first U.S. experience (with videos). Gastrointest Endosc.

[12] Sulz MC, Bertolini R, Frei R, Semadeni GM, Borovicka J, Meyenberger C (2014). Multipurpose use of the over-the-scope-clip system (“Bear claw”) in the gastrointestinal tract: Swiss experience in a tertiary center. World J Gastroenterol.

[13] Law R, Wong Kee Song LM, Irani S, Baron TH (2015). Immediate technical and delayed clinical outcome of fistula closure using an over-the-scope clip device. Surg Endosc.

[14] Heinrich H, Gubler C, Valli PV (2017). Over-the-scope-clip closure of long lasting gastrocutaneous fistula after percutaneous endoscopic gastrostomy tube removal in immunocompromised patients: a single center case series. World J Gastrointest Endosc.

[15] Alshafei A, Deacy D, Antao B (2017). Risk factors for a persistent gastrocutaneous fistula following gastrostomy device removal: a tertiary center experience. J Indian Assoc Pediatr Surg.

[16] Abraham A, Vasant DH, McLaughlin J, Paine PA (2015). Endoscopic closure of a refractory gastrocutaneous fistula using a novel over-the-scope Padlock clip following de-epithelialisation of the fistula tract. BMJ Case Rep.

[17] Hameed H, Kalim S, Khan YI (2009). Closure of a nonhealing gastrocutanous fistula using argon plasma coagulation and endoscopic hemoclips. Can J Gastroenterol.

[18] Gay-Chevallier S, Lupu A, Rivory J (2019). Closure of non-healing gastrocutaneous fistula after percutaneous endoscopic gastrostomy by endoscopic submucosal dissection and over-the-scope clip. Endoscopy.

[19] Pausawasdi N, Angkurawaranon C, Chantarojanasiri T (2020). Successful closure of a benign refractory tracheoesophageal fistula using an over-the-scope clip after failed esophageal stent placement and surgical management. Clin Endosc.

